# Social cohesion, trust, and utilisation of maternal health services among refugee and host community women in Bangladesh and Kenya

**DOI:** 10.1111/disa.70001

**Published:** 2025-08-01

**Authors:** HaEun Lee, Ryan Rego, Neyat Fiseha, Ashok Kumar Barman, Nimo Sharif, Peninah Wachira, Joe Kolars, Akbar Waljee, Rubhana Rakqib, Md Sirajul Islam, Amina Abubakar

**Affiliations:** ^1^ University of Michigan School of Nursing United States; ^2^ Clinton Health Access Initiative United States; ^3^ University of Michigan Medical School United States; ^4^ International Centre for Diarrheal Disease Research Bangladesh; ^5^ Institute for Human Development, The Aga Khan University Kenya; ^6^ Faculty of Medicine and Health Sciences, University of Antwerp Belgium; ^7^ Center for Global Health Equity, University of Michigan United States; ^8^ Learning Health Sciences, University of Michigan Medical School United States

**Keywords:** Bangladesh, host communities, host–refugee trust, Kenya, maternal health care, refugee communities, social cohesion

## Abstract

The global refugee crisis places significant pressure on host communities, particularly in low‐ and middle‐income countries. Social cohesion and trust between refugee and host communities are critical for maternal health service utilisation. This study explores the relationship between host–refugee trust, social cohesion, and maternal health service use in Bangladesh and Kenya, focusing on facility‐based delivery, antenatal care visits, and postnatal care attendance. To do so, a cross‐sectional survey was administered among 649 refugee/displaced women and 371 host community women in the two locations. The results show high trust levels in both groups, while social cohesion is greater among refugee/displaced women. A higher social cohesion score is significantly associated with increased odds of attending four or more PNC appointments (adjusted odds ratio: 1.03; 95 per cent confidence interval: 1.01–1.05). Strengthening social cohesion may enhance maternal health service utilisation, especially postnatal care, in refugee‐hosting settings, underlining the need for interventions fostering community bonds.

## 1 INTRODUCTION

As of the end of 2023, an estimated 117.3 million people worldwide, or one in 69 individuals, were forcibly displaced (UNHCR, 2024b). Approximately 75 per cent of these displaced persons are hosted by low‐ and middle‐income countries (LMICs), with the least developed nations providing asylum to 21 per cent of the total (UNHCR, 2024b). While hosting displaced populations in LMICs is crucial for safeguarding their rights and well‐being, it imposes significant pressure on the limited resources of these states and can create complex social, economic, and political dynamics between host communities and displaced populations.

The 1951 Refugee Convention (United Nations, 1951) establishes the right of refugees to access healthcare, including maternal health services, in signatory countries. The practical implementation of this right depends, however, on several factors, including the national health system, government policies, humanitarian support, and the role of non‐governmental organisations (NGOs). This study examines maternal health service access across four distinct settings—Mirpur in Dhaka, Bangladesh; Leda (Camp 24) in Teknaf, Cox's Bazar, Bangladesh; Eastleigh in Nairobi, Kenya; and Dadaab refugee camp, Kenya—each with varying healthcare infrastructure, policy frameworks, and levels of government and nongovernmental support. Further details on these settings are provided in the study setting subsection (2.1).

The impact of refugee hosting on host populations has long been debated. Meyer et al. (2011) reviewed how health service delivery in long‐term refugee situations affects health service utilisation by refugees and host populations. They found that hosting refugees can be both a burden and a benefit, depending on factors such as the relationship between host and displaced populations and the broader social, economic, and political context. The health outcomes of and access to services by displaced populations can sometimes exceed those of host community members, potentially causing tension between the groups (Meyer et al., 2011; Davidson et al., 2022). This disparity may be due to factors such as closer proximity to health facilities, access to food and non‐food rations, and free services in camp‐based settings (Hynes et al., 2002; Meyer et al., 2011).

Regarding maternal health, several studies have indicated that refugees often experience better access to such services and outcomes as compared to host communities owing to emergency obstetric services, better‐resourced health facilities, and free transportation to referral hospitals provided by governmental entities and NGOs (Hynes et al., 2002; Orach and De Brouwere, 2004; Orach, Dubourg, and De Brouwere, 2007). Yet, a review by the World Health Organization (WHO) of the common health needs of refugees and migrants highlights that the refugee population, despite variations within it, generally experiences poorer reproductive health outcomes as compared to women in the host community population (WHO, 2021). This disparity is reflected in higher rates of maternal and neonatal morbidity and mortality, largely driven by financial constraints, language and communication barriers, real or perceived discrimination, limited access to health information, and stigma associated with seeking reproductive healthcare services.

This study examines the utilisation of maternal health services among refugee and host community women by assessing facility‐based delivery, antenatal care (ANC) visits, and postnatal care (PNC) attendance. Facility‐based deliveries, as compared to home births, have been shown to improve maternal and child health outcomes by ensuring access to skilled healthcare providers and the necessary resources to identify and manage promptly complications during labour and delivery (Campbell and Graham, 2006). Early and regular ANC visits are equally critical, as they help to monitor foetal growth, detect infections, support the survival of newborns, and promote positive health‐seeking behaviours (Campbell and Graham, 2006; WHO, 2017). Similarly, attending all four PNC appointments is essential for pinpointing postnatal danger signs, supporting postpartum family planning decisions, encouraging exclusive breastfeeding, and educating mothers about neonatal warning signs (WHO, 2017).

Social cohesion and trust may play a crucial role in shaping maternal health service utilisation. Social cohesion, while related to trust, extends beyond it by fostering a sense of shared purpose and cooperation among different groups (Betts et al., 2022). Literature shows it to be a critical social determinant of health (Miller et al., 2020), including overall psychological health (Choi and Matz‐Costa, 2018), higher use of condoms (Duff et al., 2015), and all‐cause mortality (Inoue et al., 2013). Although social cohesion is less studied in the context of maternal health, research suggests it serves as a protective factor in mitigating women's pain during pregnancy (Yamada et al., 2021). Furthermore, a systematic review found that women in communities with higher social capital and cohesion were significantly more likely to use ANC services, highlighting the influence of emotional support and health information from trusted individuals on the use of maternal and child health services (Mengesha et al., 2021). Similarly, social cohesion between refugee women and the host community—as well as mutual trust within and between these groups—can significantly impact the flow of information and its trustworthiness, as well as emotional and peer support. These dynamics, in turn, may shape the perceptions of local healthcare providers and services, ultimately affecting maternal health service utilisation and outcomes (Yeo et al., 2023).

To the best of our knowledge, however, there is no study that appraises the association between host–refugee trust, social cohesion, and utilisation of maternal health services. Rather, available research frequently analyses refugee women's trust in providers and health systems as opposed to trust in the host community at large (Hawkins et al., 2021; Rustad et al., 2021; Kasper et al., 2022). In this study, trust was assessed by measuring participants' trust in other refugee/displaced individuals and members of the host community. Social cohesion, meanwhile, was measured using participants' perceptions of their neighbourhood and broader community connections, without distinguishing whether these linkages were primarily with refugee/displaced individuals or members of the host community.

In refugee‐hosting settings, strong social cohesion can facilitate better integration of refugees, which is essential for effective health service utilisation (Betts et al., 2022). While previous studies have examined either social cohesion and its relationship with refugee/displaced populations or the association between social cohesion and refugee health, few have simultaneously captured both refugees' and hosts' trust in each other, social cohesion, and health service utilisation (Olney, Badiuzzaman, and Hoque, 2019; Betts et al., 2022; Ermansons et al., 2023; Pfarrwaller and Suris, 2024). For example, Betts et al. (2022) found that increased positive interactions between refugees and host community members led to more favourable perceptions of each other. Pfarrwaller and Suris (2024) also revealed that social support and family cohesion act as protective factors for both physical and mental health among young migrants and refugees.

This study seeks to fill this gap by examining the association between host–refugee trust, social cohesion, and utilisation of maternal health services, focusing on facility‐based delivery, ANC visits, and PNC attendance, in four locations within Bangladesh and Kenya. We hypothesise that high levels of host–refugee trust and social cohesion will be associated with higher utilisation of maternal health services.

## 2 METHODS

Cross‐sectional data were collected from four locations in two countries, as noted above: (i) Mirpur in Dhaka, Bangladesh; (ii) Leda (Camp 24) in Teknaf, Cox's Bazar, Bangladesh; (iii) Eastleigh in Nairobi, Kenya; and (iv) Dadaab refugee camp, Kenya. The original study design called for the recruitment of 400 refugee/displaced individuals and 400 host community members from each site, except for Dadaab, which had no comparable host community owing to its isolation. Of the original sample, only female participants were included in this study. The data were gathered between June and December 2022.

### 2.1 Study setting

Dhaka, the capital of Bangladesh, receives an estimated 300,000–400,000 people annually due to climate hazards (Molla et al., 2014). These internally displaced persons (IDPs), often termed ‘climate refugees’, face challenges like those confronting refugees who have crossed international borders and are included in this study for comparative purposes. They often dwell in informal settlements and experience harsh living conditions and marginalisation, resulting in poor maternal health outcomes. These women rely on both government and non‐government healthcare facilities but have lower maternal health service utilisation than non‐displaced women (Haque, Parr, and Muhidin, 2020a, 2020b). In 2022, 49 per cent of women in Dhaka attended four or more ANC appointments, 70 per cent delivered in a health facility, and 58 per cent received postnatal care within two days—exceeding the national averages of 65, 58, and 55 per cent, respectively (NIPORT, Medical Education and Family Welfare Division, and MOHFW, 2024).

Cox's Bazar is the largest refugee camp in the world, hosting more than one million Rohingya refugees, an ethnic Muslim minority from Myanmar that has been denied citizenship (UNHCR, 2024a). While the initial exodus of Rohingya refugees to Cox's Bazar region dates to 1784, most Rohingya refugees, approximately 745,000, arrived in August 2017, fleeing severe persecution and violence (Hasan, Hossain, and Atar, 2024). There are now believed to be approximately one million Rohingya refugees in Bangladesh, which is probably an underestimation, making them the world's largest stateless population (UNHCR, n.d.a.). The camp has 58 health posts, 46 primary healthcare centres, and one facility with comprehensive emergency obstetric and newborn care (WHO, 2024). While 76 per cent of the refugee population has access to birthing services and 57.7 per cent to ANC services, challenges to accessing them include distance, transportation barriers, and security risks (UNHCR, 2023; Hasan, Hossain, and Atar, 2024).

Eastleigh, an urban neighbourhood of Nairobi, hosts a large Somali refugee population, estimated to make up approximately 20 per cent of its residents (Im et al., 2017). Refugee women must navigate Kenya's public health system, which, despite offering free maternity services, presents financial and documentation barriers (Jemutai et al., 2021). Public hospitals like Pumwani Maternity Hospital serve refugees, but high costs, fear of discrimination, and cultural preferences push many of these women towards community‐based Somali clinics or home births (Lusambili et al., 2020). NGOs such as the International Organization for Migration and the International Rescue Committee (IRC) offer some subsidised maternal care, but access remains inconsistent (IRC, n.d.; IOM, n.d.).

Dadaab, one of the world's largest refugee camps, is home to more than 300,000 refugees, mostly Somalis (Negash and Camilo de Sales, 2024). Healthcare services are coordinated by the United Nations High Commissioner for Refugees (UNHCR) and provided free of charge by partners such as the IRC, Kenya Red Cross, and Médecins Sans Frontières (MSF, 2023). The camp has 16 health posts, four hospitals, and a maternity centre, alongside the provision of community outreach services; however, security threats have led to increased restrictions, complicating aid delivery and access to care (Gee, Vargas, and Foster, 2019).

The study sites were selected based on their significance in hosting large numbers of displaced people, both IDPs and refugees. In Bangladesh, Cox's Bazar was until recently the only refugee camp and is the largest in the world (UNHCR, n.d.b.). Given its scale and importance, as well as the presence of an adjacent host community, it was chosen as a study site. In Dhaka, the Mirpur area was selected for convenience, as our collaborator, the International Centre for Diarrhoeal Diseases Research, Bangladesh, already has an established presence there. While Mirpur was the chosen site, we suspect that conditions in other informal settlements in Dhaka are comparable. In Kenya, Dadaab was selected because it is the largest refugee camp in the country and among the largest in the world. In Nairobi, Eastleigh was chosen because it has the highest concentration of refugees in the city (Abdi, 2010).

### 2.2 Sample

The original study design employed the Expanded Program on Immunization's sampling method, entailing the selection of clusters with probability proportional to the most recent census estimates and systematic selection from a cumulative population list (WHO, 2008). The study included 2,858 participants, with 1,239 from Kenya and 1,619 from Bangladesh. Following the exclusion of male participants and those that did not have a child aged five years or younger, the total was reduced to 1,020 women: 750 from Bangladesh and 270 from Kenya. In Bangladesh, there were 267 refugee/displaced women and 197 host community women in Dhaka, while in Cox's Bazar, there were 214 refugee/displaced women and 72 host community women. In Kenya, there were 51 refugee/displaced women and 102 host community women in Eastleigh, while in Dadaab, there were 117 refugee/displaced women.

Each day, research assistants fluent in local languages conducted household interviews. After confirming eligibility based on criteria outlined in Table 1, the participants provided informed consent before completing a two‐hour survey. The surveys were administered according to participants' literacy levels, with the option for questions and answers to be read aloud if needed. The details of the sampling method and data collection can be found in Rego et al. (2025).

**TABLE 1:** Inclusion and exclusion criteria for the study's participants in each category and location.

Inclusion criteria	Exclusion criteria
*Refugee community, Cox's Bazar, Bangladesh*	
Woman*	Man
Survey participant aged 18 years or older; capable of consenting	Does not consent
All household members have lived in Cox's Bazar for at least one year (unless under one year of age, in which case since birth)	–
Is a forcibly‐displaced Myanmar national	–
Have a child who is currently five years old or younger*	Youngest child is more than five years old
*Host community, Cox's Bazar, Bangladesh*	
Woman*	Man
Survey participant aged 18 years or older; capable of consenting	Does not consent
All household members have lived in Teknaf for at least one year (unless under one year of age, in which case since birth)	Is not a Bangladeshi national
Have a child who is currently five years old or younger*	Youngest child is more than five years old
*Refugee community, Dhaka, Bangladesh*	
Woman*	Man
Survey participant aged 18 years or older; capable of consenting	Does not consent
All permanent household members have lived in Mirpur for at least one year (unless under one year of age, in which case since birth)	–
Relocated to Dhaka owing to climate change	–
Have a child who is currently five years old or younger*	Youngest child is more than five years old
*Host community, Dhaka, Bangladesh*	
Woman*	Man
Survey participant aged 18 years or older; capable of consenting	Does not consent
All permanent household members have lived in Dhaka for at least one year (unless under one year of age, in which case since birth)	Relocated to Mirpur owing to climate change‐related disaster
Have a child who is currently five years old or younger*	Youngest child is more than five years old
*Refugee community, Eastleigh, Kenya*	
Woman*	Man
Survey participant aged 18 years or older; capable of consenting	Does not consent
Somali ethnic group	Not a refugee
All household members have lived in Eastleigh for at least one year (unless under one year of age, in which case since birth)	–
Have a child who is currently five years old or younger*	Youngest child is more than five years old
*Host community, Eastleigh, Kenya*	
Woman*	Man
Survey participant aged 18 years or older; capable of consenting	Does not consent
All household members are non‐refugees	–
Somali ethnic group	–
All household members have lived in Eastleigh for at least one year (unless under one year of age, in which case since birth)	–
Have a child who is currently five years old or younger*	Youngest child is more than five years old
*Refugee community, Dadaab, Kenya*	
Woman*	Man
Survey participant aged 18 years or older; capable of consenting	Does not consent
No one in the household has a Kenyan identification (ID) card	–
Somali ethnic group	–
All household members have lived in Dadaab for at least one year (unless under one year of age, in which case since birth)	–
Have a child who is currently five years old or younger*	Youngest child is more than five years old

**Note:** * Criteria used for data extraction, not original sampling.

**Source:** authors.

### 2.3 Measures

Independent variables were trust in host population, trust in refugee/displaced population, and perceived social cohesion, whereas dependent variables were facility‐based delivery, making four or more ANC visits, and attending four or more PNC appointments. Demographic variables were age, education level, marital status, household income, years lived in current setting, and number of children under five years old.

Host–refugee trust was assessed using two statements, ‘I trust the Bangladeshis/Kenyans in my community’ and ‘I trust the refugee/displaced people in my community’, rated on a five‐point Likert scale. Responses were dichotomised into ‘yes’ (strongly agree/agree) and ‘no’ (neutral/disagree/strongly disagree). While trust is a complex concept, national (Taylor, Funk, and Clark, 2007; Vinck and Pham, 2015) and international (Buzasi, 2015) surveys frequently measure general trust in a specific people group through a one‐item question.

Social cohesion was measured using Buckner's (1988) Neighborhood Cohesion Instrument, consisting of 18 items rated on a five‐point Likert scale, with the highest possible score being 80. Items like ‘Overall, I am very attracted to living in this neighbourhood’ and ‘I feel like I belong to this neighbourhood’ were included, with inversely worded items subtracted from the total score. The full questionnaire is presented in Table 2.

**TABLE 2:** Questionnaire used to assess social cohesion.

Question	Score
Overall, I am very attracted to living in this neighbourhood	5 = Strongly agree 4 = Agree 3 = Neutral 2 = Disagree 1 = Strongly disagree
2 I feel like I belong to this neighbourhood	5 = Strongly agree 4 = Agree 3 = Neutral 2 = Disagree 1 = Strongly disagree
3 I visit my neighbours in their homes	5 = Strongly agree 4 = Agree 3 = Neutral 2 = Disagree 1 = Strongly disagree
4 The friendships and associations I have with other people in my neighbourhood mean a lot to me	5 = Strongly agree 4 = Agree 3 = Neutral 2 = Disagree 1 = Strongly disagree
5 Given the opportunity, I would like to move out of this neighbourhood*	5 = Strongly agree 4 = Agree 3 = Neutral 2 = Disagree 1 = Strongly disagree
6 If the people in my neighbourhood were planning something, I'd think of it as ‘we’ were doing rather than ‘they’ were doing	5 = Strongly agree 4 = Agree 3 = Neutral 2 = Disagree 1 = Strongly disagree
7 If I needed advice about something I could go to someone in my neighbourhood	5 = Strongly agree 4 = Agree 3 = Neutral 2 = Disagree 1 = Strongly disagree
8 I think I agree with most people in my neighbourhood about what is important in life	5 = Strongly agree 4 = Agree 3 = Neutral 2 = Disagree 1 = Strongly disagree
9 I believe my neighbours would help me in an emergency	5 = Strongly agree 4 = Agree 3 = Neutral 2 = Disagree 1 = Strongly disagree
10 I feel loyal to people in my neighbourhood	5 = Strongly agree 4 = Agree 3 = Neutral 2 = Disagree 1 = Strongly disagree
11 I borrow things and exchange favours with my neighbours	5 = Strongly agree 4 = Agree 3 = Neutral 2 = Disagree 1 = Strongly disagree
12 I would be willing to work together with others on something to improve my neighbourhood	5 = Strongly agree 4 = Agree 3 = Neutral 2 = Disagree 1 = Strongly disagree
13 I plan to remain a resident of this neighbourhood for a number of years	5 = Strongly agree 4 = Agree 3 = Neutral 2 = Disagree 1 = Strongly disagree
14 I like to think of myself as similar to the people who live in this neighbourhood	5 = Strongly agree 4 = Agree 3 = Neutral 2 = Disagree 1 = Strongly disagree
15 I rarely have neighbours over to my house*	5 = Strongly agree 4 = Agree 3 = Neutral 2 = Disagree 1 = Strongly disagree
16 A feeling of fellowship runs deep between me and other people in this neighbourhood	5 = Strongly agree 4 = Agree 3 = Neutral 2 = Disagree 1 = Strongly disagree
17 I regularly stop and talk with people in my neighbourhood	5 = Strongly agree 4 = Agree 3 = Neutral 2 = Disagree 1 = Strongly disagree
18 Living in this neighbourhood gives me a sense of community	5 = Strongly agree 4 = Agree 3 = Neutral 2 = Disagree 1 = Strongly disagree

**Note:** * Inversely worded items subtracted from the total score.

**Source:** authors, based on Buckner (1988).

Facility‐based delivery was determined by the location of the most recent instance of childbirth, categorising deliveries at government, private, or NGO facilities as ‘utilised’ and those at home or with traditional healers as ‘not utilised’. The number of ANC and PNC visits was assessed by asking about the most recent case of pregnancy, with four or more visits in each category being classified as ‘utilised’. Although the most recent WHO (2016) guidelines on ANC suggest eight or more sessions, as compared to the previous recommendation of four or more, we used the latter since the latest advice has not been widely adopted in the study settings. These three issues required the participant to reflect on their most recent delivery.

### 2.4 Analysis

Descriptive statistics were analysed using counts and percentages for categorical variables and means and standard deviations (SDs) for continuous variables. Binary logistic regression models were used to assess the relationship between independent variables (host–refugee trust and social cohesion) and dependent variables (facility‐based delivery, ANC visits, and PNC attendance) both unadjusted and adjusted for location and refugee status, age, education level, marital status, household income, number of years lived in current setting, and number of children under five years old. Adjusted odds ratios (AORs) and 95 per cent confidence intervals (CIs) were reported. Data analysis was performed using Stata 19.

### 2.5 Ethics

Ethical approval was obtained from The Aga Khan University, Nairobi (registration number: 2022/ISERC_32(v2)), the Kenya National Commission for Science, Technology and Innovation (reference number: NACOSTI/P/22/19914), and the Ethical Review Committee of the International Centre for Diarrheal Disease Research, Bangladesh (reference number: PR‐22042).

## 3 RESULTS

Table 3 presents descriptive statistics for the 1,020 women across the four locations, categorised by status: 649 refugee/displaced women and 371 host community women. The locations, as noted, are Dhaka with 267 refugee/displaced women and 197 host community women, Cox's Bazar with 214 refugee/displaced women and 72 host community women, Eastleigh with 51 refugee/displaced women and 102 host community women, and Dadaab with 117 refugee/displaced women. The average age of the participants was 29.3 years (SD: 7.9), with host community women being slightly older. Almost two‐thirds (62.2 per cent) had primary education, with host community women generally better educated. The vast majority (92.1 per cent) were married, with higher divorce/separation rates in Kenya as compared to Bangladesh. More than one‐half (55.4 per cent) of the participants had lived in their respective settings for in excess of 10 years, with refugee/displaced women generally residing longer than host community women across the three comparable locations; however, a higher proportion of host community women had lived in the setting since birth in these locations overall. Host community women reported higher household incomes, ranging from 1.4 to 3.7 times that of refugee/displaced women. All women had at least one child under the age of five.

**TABLE 3:** Sociodemographic characteristics and independent and dependent characteristics of participants stratified by refugee status and location.

	Total	Dhaka, Bangladesh		Cox's Bazar, Bangladesh		Eastleigh, Kenya		Dadaab refugee camp, Kenya
		Refugee/ displaced	Host	Refugee/ displaced	Host	Refugee/ displaced	Host	Refugee/ displaced
	N = 1,020	N = 267	N = 197	N = 214	N = 72	N = 51	N = 102	N = 117
Age, mean (SD)	29.3 (7.9)	27.2 (6.3)	28.2 (6.9)	27.7 (7.5)	28.5 (6.3)	32.3 (7.9)	34.7 (8.1)	33.4 (9.9)
Education, n (%)								
None	122 (12.0%)	0 (0.0%)	0 (0.0%)	0 (0.0%)	0 (0.0%)	25 (49.0%)	25 (24.5%)	72 (61.5%)
Primary	634 (62.2%)	265 (99.3%)	139 (70.6%)	136 (63.6%)	11 (15.3%)	8 (15.7%)	46 (45.1%)	29 (24.8%)
Secondary	210 (20.6%)	2 (0.7%)	56 (28.4%)	70 (32.7%)	31 (43.1%)	13 (25.5%)	22 (21.6%)	16 (13.7%)
Higher than secondary	54 (5.3%)	0 (0.0%)	2 (1.0%)	70 (32.7%)	30 (41.7%)	5 (9.8%)	9 (8.8%)	0 (0.0%)
Marital status, n (%)								
Married/cohabiting	939 (92.1%)	246 (92.1%)	193 (98.0%)	211 (98.6%)	67 (93.1%)	44 (86.3%)	78 (76.5%)	100 (85.5%)
Divorced/separated	58 (5.7%)	18 (6.7%)	4 (2.0%)	1 (0.5%)	2 (2.8%)	3 (5.9%)	18 (17.6%)	12 (10.3%)
Never married	23 (2.3%)	3 (1.1%)	0 (0.0%)	2 (0.9%)	3 (4.2%)	4 (7.8%)	6 (5.9%)	5 (4.3%)
Household income, mean (SD)^a^	16,771.6 (19557.7)	3,874.9 (3185.1)	14,624.6 (12275.5)	17,387.9 (12378.8)	43,541.7 (26917.7)	28,813.7 (16789.6)	41,495.1 (27168.2)	5,413.3 (5067.9)
Years lived in your current location, n (%)								
Less than one year	15 (1.5%)	1 (0.4%)	0 (0.0%)	5 (2.3%)	9 (12.5%)	0 (0.0%)	0 (0.0%)	0 (0.0%)
Between one and five years	182 (17.8%)	31 (11.6%)	56 (28.4%)	41 (19.2%)	14 (19.4%)	15 (29.4%)	18 (17.6%)	7 (6.0%)
Between five and 10 years	171 (16.8%)	13 (4.9%)	47 (23.9%)	44 (20.6%)	18 (25.0%)	9 (17.6%)	26 (25.5%)	14 (12.0%)
More than 10 years	565 (55.4%)	222 (83.1%)	88 (44.7%)	112 (52.3%)	22 (30.6%)	17 (33.3%)	17 (16.7%)	87 (74.4%)
Entire life	87 (8.5%)	0 (0.0%)	6 (3.0%)	12 (5.6%)	9 (12.5%)	10 (19.6%)	41 (40.2%)	9 (7.7%)
Number of children under five, mean (SD)	1.4 (0.7)	1.4 (0.6)	1.3 (0.5)	1.1 (0.3)	1.1 (0.4)	1.5 (0.8)	1.4 (0.7)	1.9 (1.0)
Trust host, n (%)	978 (95.9%)	264 (98.9%)	196 (99.5%)	210 (98.1%)	72 (100.0%)	45 (88.2%)	84 (82.4%)	107 (91.5%)
Trust refugees, n (%)	952 (93.3%)	264 (98.9%)	195 (99.0%)	205 (95.8%)	54 (75.0%)	44 (86.3%)	78 (76.5%)	112 (95.7%)
Neighborhood Cohesion Instrument, mean (SD)	57.5 (9.2)	62.0 (7.9)	61.9 (7.0)	56.1 (8.3)	43.1 (11.8)	57.0 (8.4)	54.0 (7.6)	55.5 (3.8)
Delivery at health centre, n (%)	675 (66.2%)	161 (60.3%)	88 (44.7%)	96 (44.9%)	65 (90.3%)	49 (96.1%)	102 (100.0%)	114 (97.4%)
Four or more antenatal care visits, n (%)	675 (68.7%)	221 (95.7%)	151 (77.0%)	96 (44.9%)	62 (86.1%)	33 (64.7%)	33 (32.4%)	79 (67.5%)
Four or more postnatal care visits, n (%)	227 (23.1%)	60 (26.0%)	11 (5.6%)	13 (6.1%)	32 (44.4%)	28 (54.9%)	25 (24.5%)	58 (49.6%)

**Note**: * The *p*‐values were dropped from this table because they were all significant (0.001).

**Source:** authors.

Overall, 95.9 per cent of all women expressed trust in the host community, and 93.3 per cent expressed trust in the refugee/displaced community. Yet, host community women generally reported lower trust in the refugee/displaced community as compared to refugee/displaced women. Social cohesion scores were higher among refugee/displaced women as compared to host community women across all three settings (excluding Dadaab as there is no host community data for that site).

Dhaka had high trust in the host community, refugee/displaced community, and social cohesion across both sample groups as compared to samples in all locations. In Cox's Bazar, host community women reported lower trust in the refugee/displaced community (75.0 per cent) as compared to refugee/displaced women (95.8 per cent), although trust in the host community remained high across both groups. Social cohesion scores were also higher among refugee/displaced women (56.1; SD: 8.3) than host women (43.1; SD: 11.8).

Maternal health service utilisation patterns differed between Dhaka and Cox's Bazar. In the capital, host community women had lower rates of facility‐based delivery (44.7 per cent), four or more ANC visits (77.0 per cent), and four or more PNC appointments (5.6 per cent) as compared to refugee/displaced women (60.3, 95.7, and 26 per cent, respectively). In contrast, in Cox's Bazar, refugee/displaced women had lower rates of facility‐based delivery (44.9 per cent), four or more ANC visits (44.9 per cent), and four or more PNC appointments (6.1 per cent) as compared to host community women (90.3, 86.1, and 44.4 per cent, respectively).

In Eastleigh, refugee/displaced women reported higher trust in both the host community and the refugee/displaced community than host community women (88.2 versus 82.4 per cent and 86.3 versus 76.5 per cent, respectively). Social cohesion scores were also higher among refugee/displaced women (57.0; SD: 8.4) as compared to host community women (54.0; SD: 7.6). In Dadaab, 91.5 per cent of refugee/displaced women trusted the host community, 95.7 per cent trusted fellow refugees/displaced individuals, and the neighbourhood cohesion score was 55.5 (SD: 3.8).

Maternal health service utilisation patterns in Kenya revealed relatively greater access to facility‐based delivery. In Eastleigh, all host community women delivered at a health facility, as compared to 96.1 per cent of refugee/displaced women. However, more refugee/displaced women attended four or more ANC appointments (64.7 per cent) and four or more PNC appointments (54.9 per cent) as compared to host community women (32.4 and 24.5 per cent, respectively). Refugee/displaced women in Dadaab exhibited similar trends in maternal health service utilisation as those in Eastleigh, with 97.4 per cent delivering at a health facility, 67.5 per cent attending four or more ANC appointments, and 49.6 per cent attending four or more PNC appointments.

The logistic regression results in Table 4 indicate key statistically significant relationships between social cohesion, trust, and maternal health service utilisation. Unadjusted models show that greater trust in hosts (odds ratio (OR): 0.09; 95 per cent CI: 0.02–0.39), greater trust in refugees (OR: 0.14; 95 per cent CI: 0.06–0.36), and greater social cohesion (OR: 0.95; 95 per cent CI: 0.94–0.97) are associated with lower likelihood of delivery at a health facility. But these associations are not significant in the adjusted models. Additionally, trust in hosts is associated with an increased likelihood of making four or more ANC visits (OR: 2.52; 95 per cent CI: 1.34–4.77) and trust in refugees is associated with lower odds of making four or more PNC visits (OR: 0.53; 95 per cent CI: 0.31–0.88), although these also lose significance in the adjusted model. However, neighbourhood cohesion is significantly associated with an increased likelihood of making four or more PNC visits in the adjusted model (AOR: 1.03; 95 per cent CI: 1.01–1.05), suggesting that community cohesion has a potential role in postnatal care utilisation.

**TABLE 4:** Adjusted and unadjusted association between host–refugee trust, social cohesion, and utilisation of maternal health services.

	Delivery at health centre		Four or more ANC visits		Four or more PNC visits	
	OR (95 per cent CI)	AOR (95 per cent CI)	OR (95 per cent CI)	AOR (95 per cent CI)	OR (95 per cent CI)	AOR (95 per cent CI)
Trust host	0.09 (0.02–0.39)***	0.38 (0.07–2.05)	2.52 (1.34–4.77)**	0.96(0.42–2.22)	1.04 (0.49–2.21)	1.81 (0.74–4.37)
Trust refugees	0.14 (0.06–0.36)***	0.77 (0.25–2.40)	1.59 (0.96–2.63)	1.09 (0.53–2.23)	0.53 (0.31–0.88)*	0.55 (0.28–1.09)
Neighbourhood cohesion scale	0.95 (0.94–0.97)***	0.99 (0.98–1.02)	1.01 (0.99–1.02)	0.99 (0.97–1.01)	0.99 (0.97–1.00)	1.03 (1.01–1.06)**

**Notes**: * *p* < 0.05; ** *p* < 0.01; *** *p* ≤ 0.001. AOR adjusted for location and refugee status, age, education, marital status, income, years lived in respective setting, and number of children under five.

**Source:** authors.

## 4 DISCUSSION

This study explores how host–refugee trust and social cohesion are associated with maternal health service utilisation in Bangladesh and Kenya. The findings indicate that social cohesion—rather than interpersonal trust alone—plays a more consistent role in shaping women's engagement with PNC services across both refugee/displaced and host communities. That the observed associations are strongest in relation to PNC utilisation underscores the importance of community dynamics in the postpartum period.

Our results align with previous research highlighting the role of social cohesion in promoting health service utilisation (Mengesha et al., 2021). The higher social cohesion scores among refugee/displaced women as compared to host community women suggest that a sense of belonging and support within a community can enhance access to and use of health services. This is consistent with Mengesha et al. (2021), who found that higher social capital and cohesion increased the likelihood of utilising ANC services.

Despite high levels of trust in the host community reported by both refugee/displaced and host community women, these did not translate into increased use of facility‐based delivery or ANC services. This may suggest that while trust is an important factor, it is not sufficient on its own to improve overall health service utilisation. Social cohesion, which encompasses not only trust but also a sense of community, support, and belonging, appears, as emphasised above, to be a more influential factor in accessing PNC services. Yet, these findings should be interpreted with caution, as higher trust levels in both host and refugee communities, as well as greater social cohesion, were paradoxically associated with lower facility‐based delivery rates. Although these associations were not significant after adjusting for covariates, they suggest that additional factors, such as healthcare accessibility and service delivery models, may shape maternal health service utilisation.

Maternal health service availability varied significantly across the study settings, which may explain some of the observed differences in service utilisation. In Dhaka, both internally displaced and host community women theoretically have access to government and non‐government facilities, as these services are integrated throughout the city; however, research indicates that climate‐displaced women in rural Bangladesh are significantly less likely to deliver at health centres, or make ANC and PNC visits, as compared to non‐displaced women (Haque, Parr, and Muhidin, 2020a, 2020b). Surprisingly, in our study, displaced women in Dhaka had higher rates of facility‐based delivery, four or more ANC visits, and four or more PNC visits as compared to host community women, suggesting that urban healthcare infrastructure may have mitigated some barriers.

Conversely, in Cox's Bazar, despite the presence of 58 health posts and 46 primary healthcare centres catering specifically to Rohingya refugees, obstacles such as mobility restrictions and long travel distances likely contributed to lower maternal health service utilisation among refugee women as compared to host community women (Hasan, Hossain, and Atar, 2024; WHO, 2024). It is also worth noting that this group may have not been comfortable visiting the healthcare centres owing to fears relating to relocation to the new Bhasan Char refugee camp, but this study did not explore the matter (Human Rights Watch, 2021).

In Kenya, refugee/displaced women in Eastleigh accessed ANC and PNC services more frequently than host community women, possibly because of their reliance on the national public health system, which theoretically offers free maternity services (Jemutai et al., 2021). The lack of dedicated refugee maternal health facilities, however, may have led to increased use of public health services, explaining their higher maternal healthcare utilisation. Dadaab, in contrast, has dedicated maternal health facilities for refugees provided by NGOs, yet security concerns are a significant constraint on healthcare access (Gee, Vargas, and Foster, 2019). The long‐standing presence of Somali refugees in Dadaab has contributed to a better structured and established healthcare system, whereas Cox's Bazar, despite its historical refugee presence, continues to face service delivery challenges owing to its rapid and recent expansion into a major humanitarian response setting.

The length of time in displacement may further shape social cohesion and health service utilisation. Newly‐arrived refugees often experience greater barriers in accessing healthcare as compared to those who have been displaced for extended periods (McCann et al., 2023). This is particularly relevant in Cox's Bazar: most Rohingya refugees arrived in 2017 and may still be adapting to available healthcare services. In contrast, refugees in Dadaab, who have lived in the camp for decades, may have developed stronger social networks and more established health‐seeking behaviours.

Sociocultural norms also play a significant part in shaping maternal health‐seeking behaviours. For example, Somali refugee women in Eastleigh often prefer home deliveries owing to traditional birthing practices and economic challenges related to accessing facility‐based deliveries, especially since the COVID‐19 (coronavirus disease 2019) pandemic (Lusambili et al., 2020). Similarly, Rohingya women in Cox's Bazar may face gender norms that restrict their mobility, affecting their ability to seek maternal healthcare (Hossain et al., 2023). Addressing these cultural barriers through community‐based interventions and culturally‐appropriate outreach efforts is essential for improving maternal health service uptake.

Levels of social cohesion varied between the study sites. In Dhaka, social cohesion, including inter‐ and intra‐group trust, was high among both displaced and host communities. In Cox's Bazar, social cohesion was higher among refugee/displaced women than host community women. Similarly, in Eastleigh, refugee/displaced women exhibited greater social cohesion than host community women, although the differences were less pronounced. These variations may be partially explained by differences in residential settings and aid distribution models.

In Eastleigh and Dhaka, refugee/displaced and host communities are interspersed, allowing for greater interaction and shared access to services (Lindley, 2007; Molla et al., 2014). In contrast, Cox's Bazar and Dadaab operate under strict encampment models: refugee populations are housed in designated areas separate from host communities, probably influencing social cohesion scores and healthcare access (Vella and Rueda, 2022; Uddin, 2024).

Additionally, aid distribution models differed across the settings. In Dhaka, aid was integrated into host community services, whereas in Cox's Bazar, humanitarian assistance was largely directed towards refugees, sometimes leading to tensions between the two groups (Mohiuddin and Molderez, 2023) Such tensions, stemming from inequitable aid distribution, have been observed in several crises (Anomat Ali, Imana, and Ocha, 2017; Sturridge et al., 2024). Ensuring equitable service provision for both refugees and host communities could help to mitigate these tensions, strengthen social cohesion, and improve healthcare utilisation.

Dadaab, which hosts a predominantly Somali Muslim refugee population, presents an opportunity for comparative analysis with Cox's Bazar. Both settings place mobility restrictions on refugees and there are ongoing tensions with local communities. Yet, maternal health service utilisation differs, potentially because of varying levels of humanitarian assistance, healthcare accessibility, and sociocultural factors. For example, while Somali refugees in Dadaab and Eastleigh share religious beliefs and cultural practices, broader contextual differences—such as Kenya being a majority Christian country while Bangladesh is predominantly Muslim—may further shape host–refugee dynamics and healthcare access (Office of International Religious Freedom, United States Department of State, 2022; Marshall, 2023). Future research should examine how religious and cultural influences intersect with policy differences in shaping maternal healthcare utilisation in these locations.

The stark difference in maternal health service utilisation between Dhaka and Cox's Bazar may be attributed to differences in service delivery models, geographical barriers, and mobility restrictions. Dhaka's urban setting offers better access to healthcare infrastructure, while Cox's Bazar's camp‐based setup isolates refugees from host community services. Kenya's refugee health services operate under both integrated and parallel health service models; UNHCR funds specific refugee services while also supporting referrals to government facilities (Burnham, Rowley, and Ovberedjo, 2003; Tuepker and Chi, 2009). Similar models exist in Bangladesh: refugees in Cox's Bazar primarily rely on parallel health services, whereas displaced populations in Dhaka access integrated government healthcare systems. Many refugee‐hosting settings use a combination of both models: camp‐based facilities provide initial care, and serious cases are referred to government facilities (Orach and De Brouwere, 2004; Rutta et al., 2005).

Our findings highlight the critical role of social cohesion in the utilisation of maternal health services. They also suggest that different health system models and aid distribution processes could significantly impact maternal health outcomes. Additionally, these results underscore the importance of establishing trust and developing social cohesion in order also to involve community members in maternal health interventions. A systematic review of the delivery of maternal and neonatal health interventions in conflict settings similarly emphasises the limited engagement of community members in maternal health interventions, as well as infrequent reporting of postnatal care—furthermore, details are often lacking about the specific components of such care (Munyuzangabo et al., 2021).

The limitations of this study include the lack of data on specific challenges to accessing maternal health services (such as distance, cost, and childcare) and the variability in geography and healthcare contexts. The differences among the four study locations also pose problems for generalisability, as variations in health systems and cultural norms may influence maternal health‐seeking behaviours. What is more, the study is subject to social desirability bias: participants may have provided socially‐acceptable responses to survey questions rather than accurate ones, potentially affecting data validity. While a significant association was found between social cohesion and PNC visits, the difference was small, requiring cautious interpretation. However, the inclusion of diverse populations is a strength of this study.

## 5 CONCLUSION

Our study highlights the potential role of social cohesion in influencing maternal health service utilisation among refugee and host community women, although the observed disparities were modest. While trust between host and refugee populations is essential, fostering social cohesion, which also includes components of trust, may be more effective in enhancing access to and use of maternal health services, particularly PNC. Future research should explore interventions aimed at strengthening social cohesion in refugee‐hosting settings and their impact on health service utilisation. Policymakers and health organisations should consider strategies that promote community integration and support to improve the maternal health outcomes of both refugee and host populations. By addressing these factors, we can better maintain the health and well‐being of displaced populations and the communities that host them.

## CONFLICT OF INTEREST STATEMENT

The authors have no financial/commercial conflicts of interest.

## FUNDING STATEMENT

This research was supported by the Center for Global Health Equity at the University of Michigan, the Office of the Director, National Institutes of Health, the National Institute of Biomedical Imaging and Bioengineering, the National Institute of Mental Health, and the Fogarty International Center (award number: U54TW012089; Amina Abubakar and Akbar K. Waljee). The content is solely the authors' responsibility and does not necessarily represent the official views of the National Institutes of Health.

## Data Availability

The data that support the findings of this study are available from the corresponding author upon reasonable request.
